# Cool and safe walks: heat stress threshold for domestic dogs living in hot and sunny regions

**DOI:** 10.1007/s00484-026-03236-y

**Published:** 2026-06-11

**Authors:** Pavlos Vinícius do Nascimento, Edilson Paes Saraiva, Tarsys Noan Silva Veríssimo, Jacinara Hody Gurgel Morais Leite, Luciana Diniz Rola, Aline Cristina Sant’Anna, Walter Esfrain Pereira, Vinícius de França Carvalho Fonsêca

**Affiliations:** 1 Research Group On Bioclimatology, Ethology and Animal Welfare, Animal Science Department, Federal University of Paraíba, Areia, Brazil; 2 Integrated College of Patos, Patos, Brazil; 3 Animal Science Department, Federal University of Paraíba, Areia, Brazil; 4 Veterinary Medicine Department, Federal University of Paraíba, Areia, Brazil; 5 Animal Science Department, São Paulo State University, Jaboticabal, Brazil; 6 Fundamental and Social Sciences Department, Federal University of Paraíba, Areia, Brazil; 7 Brain Function Research Group, Physiology Department, School of Biomedical Sciences, University of the Witwatersrand, Johannesburg, South Africa; 8 Animal Science Department, Instituto Federal Baiano, Santa Inês, Bahia Brazil

**Keywords:** Animal welfare, *Canis lupus familiaris*, Comfort, Heat injuries, TSI

## Abstract

This study proposes heat stress thresholds for dogs during outdoor walks in a hot and sunny tropical region. A total of five mixed-breed dogs had their body temperatures (from their coat, skin, paw pad and rectum) and respiratory rate measured at rest, during 12 min of walking, and during 20 min of recuperation, when water intake was recorded. A total of 135 walks were undertaken throughout the night and daytime phases, thereby exposing dogs to levels of black-globe temperature (BGT) ranging between 19 and 50 °C. Dogs kept their rectal temperature within a narrow range (38—39 °C), without signs of hyperthermia. However, walking with the dogs when the BGT was above 35 ºC resulted in substantial increments in hair-coat temperature (+ 20.5 ºC), skin temperature (+ 7.2 ºC), respiratory rate (increasing markedly, from approximately 47 to 374 breaths min⁻^1^) and water intake (+ 215 mL) compared to walks at a BGT below 30 ºC. The set of dogs’ responses exposed to different levels of radiant heat load was summarized into a single variable, the thermal stress index (TSI), with values ranging from 90 to 220. The best prediction of TSI accounted combined effects of air temperature and thermal radiation (*R*^*2*^ = 0.92). Maximum respiratory rate was recorded at TSI of 170 (Plateau = 287 breath min^−1^ or 4.78 breath s^−1^), and maximum skin temperature at TSI of 190 (Plateau = 38 ºC). In conclusion, walking dogs when TSI exceeds 170 should be approached with caution, particularly during prolonged or repeated exposure, as panting appears to become less effective beyond this threshold.

## Introduction

Dogs have occupied more space in the hearts and lives of human beings than any other pet. However, despite the mutual benefits arising from this interaction (Cook et al. 2014), they are frequently exposed to challenging environmental conditions, such as space restrictions and social isolation (Meyer et al. 2022). This issue has driven several studies that attested positive outcomes of outdoor walks on the quality of life of dogs and their owners (Westgarth et al. 2014, 2015; Yamamoto et al. 2015; Christian et al. 2016; Csoltova et al. 2017). Although daily walks provide clear benefits to dogs’ welfare, they can expose them to heat stress, which may impair thermoregulation, and in more extreme circumstances, lead to death (Bruchim et al. 2009). Based on a questionnaire survey to 624 owners of Siberian Huskies in Brazil, 30% of respondents reported that they walk their dogs at times commonly associated with high levels of radiant heat load (Veríssimo et al. 2023). In the UK, an investigation of primary veterinary clinical cases in 2016 attested that 1222 dogs had problems resulting from exposure to heat stress, with 74.5% of those related to moderate physical effort, for example, during an outdoor walk (Hall et al. 2020). Similarly, in a veterinary hospital in Israel, from a total of 54 clinical cases of dogs admitted, 50% was reported to die due to problems associated with exposure to hot conditions during moderate physical exercise (Bruchim et al. 2006).

Dogs living in tropical regions are more likely to face heat stress and its consequences during outdoor walks, as levels of solar irradiance and mean radiant temperature are high and almost constant throughout the year (Da Silva et al. 2010). Dogs are homeothermic and under certain circumstances of elevated heat load, relies primarily on panting to dissipate heat and sustain homeothermy (Hammel et al. 1958; Bruchim et al. 2017; Fonsêca et al. 2017). From the point-of-view of advances of heat strain in a dog during prolonged exposition to heat stress, panting can turn to thermal hyperventilation, a physiological state that contribute to initiate several metabolic disorders (Flournoy and Wohl 2003). A cascade of events may occur resulting from advances of heat strain, starting with alterations of the permeability of blood vessels, fluid leakage and impaired circulation, exacerbated immunological response and consequently induced systemic inflammation, ultimately resulting in multiple organ failures and death (Bruchim et al. 2017; Hall et al. 2020).

Several studies have been conducted with the aim at determining the threshold of heat stress for many domestic species, including poultry (Nawaz et al. 2021), pigs (Gómez-Prado et al. 2022), cattle (Cartwright et al. 2023), goats and sheep (Van Wettere et al. 2021; Ali et al. 2023). Mechanistic models were also developed for studying the impact of different microclimate conditions on heat-related responses of military dogs during vigorous exercise (Potter et al. 2020). However, as far as we know, there is no information on this issue for domestic dogs exposed to activities of low intensity, such as dogs taken for an outdoor walk. Recommendations in literature are not scientifically validated and is mostly based on previous determinations for other domestic species (Jordan et al. 2019). Ultimately, the majority of available heat stress models are based on simple relationship between the dry bulb temperature and humidity (Da Silva et al. 2007; Yan et al. 2020), an approach that does not represent the microclimate that an animal faces in open field conditions (Mitchell et al. 2018). Thermal radiation is the main environmental component of the heat balance of a dog, for example, during an outdoor walk in a tropical environment (Mitchell et al. 2024). Under such circumstances, dogs can be exposed to as high as 1200 W m^−2^ of solar radiation, in addition to high levels of infrared radiation emitted from the ground surface (Da Silva et al. 2010).

In this study, we aimed to determine the heat stress threshold for domestic dogs in an equatorial tropical region. To achieve this aim, heat responses were measured in mixed-breed dogs exposed to different levels of heat load at rest, during 12 min of outdoor walking, and 20 min of passive recovery. Dog’s responses during walking were then summarized into a single variable, the thermal stress index (TSI) for dogs, in order to account combined effects of air temperature, humidity, and thermal radiation.

## Materials and methods

### Location, animals and experimental design

This research was approved by the Animal Use Ethics Committee of the Federal University of Paraíba (Protocol number: 5618020621). The study was conducted in the city of Arcoverde, Brazil (08° 25′ 01″ S; 37° 03′ 30″ W; 695 m altitude) between April and September 2022. Five healthy (three females and two males), castrated, mixed breed dogs with short-haired (up to 3 cm), smooth and predominantly dark, aged between 1 and 7 years, and with mean body mass of 25.9 kg (SD = 2.3), had heat responses recorded during 135 events of outdoor walks, over a 150 day study period. During the study period, the dogs were housed in a shaded area of 20 m^2^, with water freely available. The dogs were fed at 7 AM and 7 PM with 175 g of dry food (3854 kcal/kg of metabolizable energy and 230 g/kg of crude protein), totaling 350 g per day. All dogs were selected from the NGO "Amor Animal de Arcoverde". Further details of dogs are depicted in Table 1.

**Table 1:** Characteristics of dogs and number of outdoor walks undertaken

Dogs	Sex	Age, months	Body mass, kg	Withers height, cm	Length, cm*	Number of walks, n
1	Male	48	27.7	57	67	30
2	Female	18	25.2	55	65	25
3	Male	72	23.5	53	65	25
4	Female	36	29	59	68	25
5	Female	24	24.3	54	65	30

^*^Partial body length (base of neck to base of tail)

### Outdoor walks with dogs

Before the start of data collection, in order to standardize the outdoor walks (e.g., mean speed, position of the dog in relation to the guiding person, and dog’s habituation to environmental stimuli) and to reduce dog’s reaction during procedures to collect physiological data, the dogs were trained by an experienced person during 10 consecutive days using principles of habituation and conditioning (Chiandetti et al. 2016). Commercial snacks and wet food were used as positive reinforcements. After training, the dogs underwent sessions of outdoor walks to be evaluated at rest (i.e., two minutes before starting the walk), during the 12 min walk, and during 20 min of recuperation. The time of day at which dogs were taken out for walks were randomly chosen over the study period, by covering times during morning, afternoon, and evening, between 6 AM and 11 PM (being that the sun rises at 5:30 AM and sets at 5:30 PM). A total of 135 walks were carried out, with an average duration of 12 min, when the dogs and the guiding person walked (average speed: 3.6 km h^−1^) over 725 m of concrete ground surface unshaded street, with low movement of vehicles and people. Immediately after this period, the dog was taken to the same space in which it was housed to be evaluated during a 20 min recuperation period. Each dog was only taken for a walk once a day.

### Heat responses: Respiratory rate, body temperatures and water intake

The respiratory rate (*R*_*R*_, breath min^−1^), rectal temperature (*T*_*R*_, ºC), skin temperature (*T*_*Skin*_, ºC), paw pad temperature (*T*_*Pad*_, ºC) and the hair-coat surface temperatures were recorded at rest, during the outdoor walk, and during the recuperation phase. The hair-coat surface temperatures were taken at the dorsal (*TS*_*Dorsal*_, ºC), head (*TS*_*Head*_, ºC) and flank (*TS*_*Flank*_, ºC). All measurements were taken two minutes before starting the outdoor walk, at the sixth and 12th min of the walk, and at the 10th and 20th min of the recuperation phase, a period at which dogs also had water intake (W_I_, ml animal^−1^) recorded. Two trained observers conducted the data collection, one guiding the dog and another one taking the measurements. The *R*_*R*_ was taken using video images (30 s duration) of the dog's thoracic movements, which were subsequently analyzed using *slow-motion* function (i.e., 25 frames per second). The *T*_*R*_ was obtained using a digital thermometer (Bioland, Model T104; range = 30—42 ºC; accuracy = ± 0.1 ºC; response time = 20 s) inserted ~ 3 cm into the rectum. The *T*_*Skin*_, *T*_*Pad*_ and hair-coat surface temperatures (*TS*_*Dorsal*_, *TS*_*Head*_ and *TS*_*Flank*_) were obtained using an infrared thermometer (Fluke, Model TiX500, 5689 Hz; range: −40—800 ºC; accuracy: ± 0.1 ºC; emissivity of the animal surface, ε = 0.98; Everett, WA, USA) at 15 cm from the animal. The *T*_*Skin*_ was obtained from the dog's ventral region, which was normally hairless; however, when necessary, an area of ~ 2 cm^2^ was shaved to expose the skin. The *T*_*Pad*_ was obtained from the paw pad of the left front; the *TS*_*Dorsal*_ was obtained by scanning the region between the scapula and the rump; the *TS*_*Head*_ was obtained by scanning the region between the ears and the forehead and *TS*_*Flank*_ was obtained by scanning the thoracic region, between the thoracic vertebrae and the sternum. Lastly, the W_I_ was obtained based on the difference between the quantify water offered and the amount of water remaining.

### Meteorological variables

The meteorological variables including ambient air temperature (*T*_*A*_, ºC), relative humidity (*R*_*H*_, %), black-globe temperature in the full sun (*BGT*_*Sun*_, ºC), black-globe temperature in the shade (*BGT*_*Shade*_, ºC) and ground surface temperature (*TGS*, ºC), were recorded every five minutes during the outdoor walking sessions. The black globes were positioned approximately 2 m from the animal’s microclimate, at a height of 55 cm from the ground surface. The *T*_*A*_ and *R*_*H*_ were recorded using a digital thermo-hygrometer (Incoterm, Model AK28; accuracy = ± 1 ºC and 1% relative humidity), positioned approximately 2 m from the animal's microclimate and protected from direct solar radiation. The black-globe temperatures were measured using a thermocouple (range = −10—60 ºC; accuracy = ± 1 ºC) inserted inside a plastic sphere (5 mm thick and 15 cm in diameter) painted matte black. One globe remained in the shade, and another one remained in an open area, exposed to the full sun. Lastly, the *TGS* was measured using an infrared thermometer (Fluke, Model TiX500, 5689 Hz; range = −40—800 ºC; accuracy = ± 0.1 ºC; emissivity of the floor surface, ε = 0.96; Everett, WA, USA) at 25 cm from the ground surface.

### Statistical analysis

All statistical analyses were performed using R 4.1.0 (R core Team 2021). Exploratory analyses were carried out to obtain mean, median and mode, in addition to dispersion measures (i.e., standard deviation and standard error mean), and to characterize the data distribution and most relevant sources of variation.

### Confirmatory models

The black-globe temperature measured near the animal is suggested to be the most appropriate metric of heat load related to the biological responses of animals (Mitchell et al. 2024). Therefore, in this study, the heat load experienced by dogs during the sessions of outdoor walks was characterized based on the black-globe temperature collected near dogs. Four classes of the black-globe temperature was grouped: first class (1): 25 °C ≥ BGT; second class (2): 30 °C ≤ BGT > 25 °C; third class (3): 35 °C ≤ BGT > 30 °C; fourth class (4): BGT > 35 °C. The heat responses *R*_*R*_, *T*_*R*_, *T*_*Skin*_, *T*_*Pad*_ and the hair-coat surface temperatures (*TS*_*Dorsal*_, *TS*_*Head*_ and *TS*_*Flank*_)—were analyzed as repeated measurements over time. Generalized mixed models were fitted to test the impact of heat load experienced at rest, during the outdoor walk, and during the recuperation phase on heat responses of dogs. The animal was assigned in the model as a random effect.

### Thermal stress index for dogs (TSI)

The impact of heat load conditions on the heat responses along the outdoor walk was summarized in a single variable, the TSI, by using non-categorical principal component analysis (Da Silva et al. 2014). First, based on the correlation matrix of the physiological variables (*y*_*ij*_ = *R*_*R*_, *T*_*R*_, *T*_*Skin*_, *T*_*Pad*_, *TS*_*Dorsal*_ and *TS*_*Head*_) and eigenvectors of the component with the highest eigenvalue, the first principal component (PC_1_) was used to reduce the physiological responses of dogs during the walk in a single response variable, the TSI:

$$\begin{aligned}TSI= TSkin\left(0.428\right)+TR\left(0.337\right)\\+RR\left(0.4202\right)+TSDorsal\left(0.4278\right)+\\TSHead\left(0.4140\right)+TPad(0.4142)\end{aligned}$$

where *T*_*Skin*_ is the skin temperature, *T*_*R*_ is the rectal temperature, *R*_*R*_ is the respiratory rate, *TS*_*Dorsal*_ and *TS*_*Head*_ are the hair-coat surface temperatures, and *T*_*Pad*_ is the paw pad temperature. The best fit for predicting the TSI as a function of meteorological conditions experienced along the outdoor walk was determined using multiple regression models and was chosen based on the highest coefficient of determination. The following model was fitted with *R*^*2*^ of 0.92:

$$\begin{aligned}TSI= \left(1.2779\right)+BGTSun\left(0.1541\right)-\\BGTShade\left(1.2365\right)+TA\left(5.5746\right)-\\RH\left(0.2965\right)+TGS(1.6021)\end{aligned}$$

where *BGT*_*Sun*_ and *BGT*_*Shade*_ are the temperatures of the black-globe in the sun and shade, respectively, *T*_*A*_ is the ambient air temperature, *R*_*H*_ is the relative humidity and *TGS* is the ground surface temperature. By using the linear_plateau procedure from the soiltestcorr R package, segmented regression models (broken-line regression) were fitted to determine critical levels of TSI for the dogs during the outdoor walk.

## Results

### Heat responses of dogs

The black-globe temperature in the sun averaged 31.9 ± 7.8 °C (range: 19–50 °C), and black-globe temperature in the shade averaged 26.0 ± 3.1 °C (range: 19–33 °C). Ground surface temperature averaged 27.9 ± 4.8 °C (range: 20.1–47.2 °C). During the study period, ambient air temperature averaged 25.4 ± 2.9 °C (range: 19–32 °C), and relative humidity averaged 77 ± 14.1% (range: 39–99%). The respiratory rate averaged 206 ± 65.3 breaths min⁻^1^, rectal temperature 39.0 ± 0.4 °C, and skin temperature 35.0 ± 1.5 °C.

**Fig. 1:**
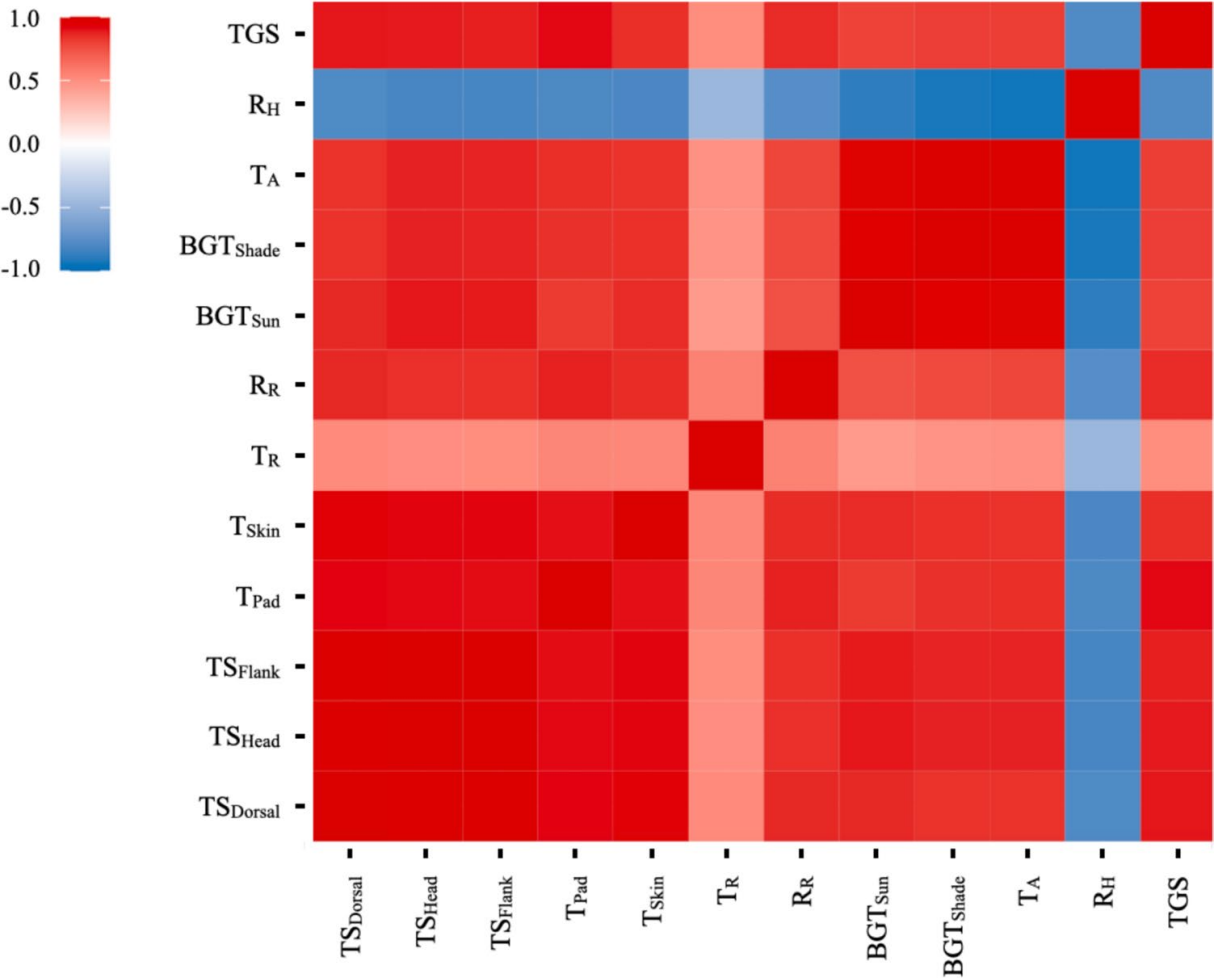
Correlogram of meteorological variables and physiological measurements of dogs (n = 5) taken during the walk and after, at rest. Ground surface temperature (TGS), relative humidity (R_H_), ambient air temperature (T_A_), black-globe temperature in the shade (BGT_Shade_), black-globe temperature in the sun (BGT_Sun_), respiratory rate (R_R_), rectal temperature (T_R_), skin temperature (T_Skin_), paw pad temperature (T_Pad_), and hair-coat surface temperatures of the flank (TS_Flank_), head (TS_Head_), and dorsal region (TS_Dorsal_)

The respiratory rate and body temperatures showed strong and positive associations with meteorological variables (Fig. 1). The respiratory rate was strongly correlated with black-globe temperature in the sun (r = 0.75), black-globe temperature in the shade (r = 0.77), ambient air temperature (r = 0.79), and ground surface temperature (r = 0.86). The paw pad temperature was strongly correlated with ground surface temperature (r = 0.91), while the hair-coat dorsal temperature was strongly correlated with black-globe temperature in the sun (r = 0.87). The rectal temperature had moderate and positive correlation with metereological variables (black-globe temperature in the sun: r = 0.58; black-globe temperature in the shade: r = 0.65).

**Fig. 2:**
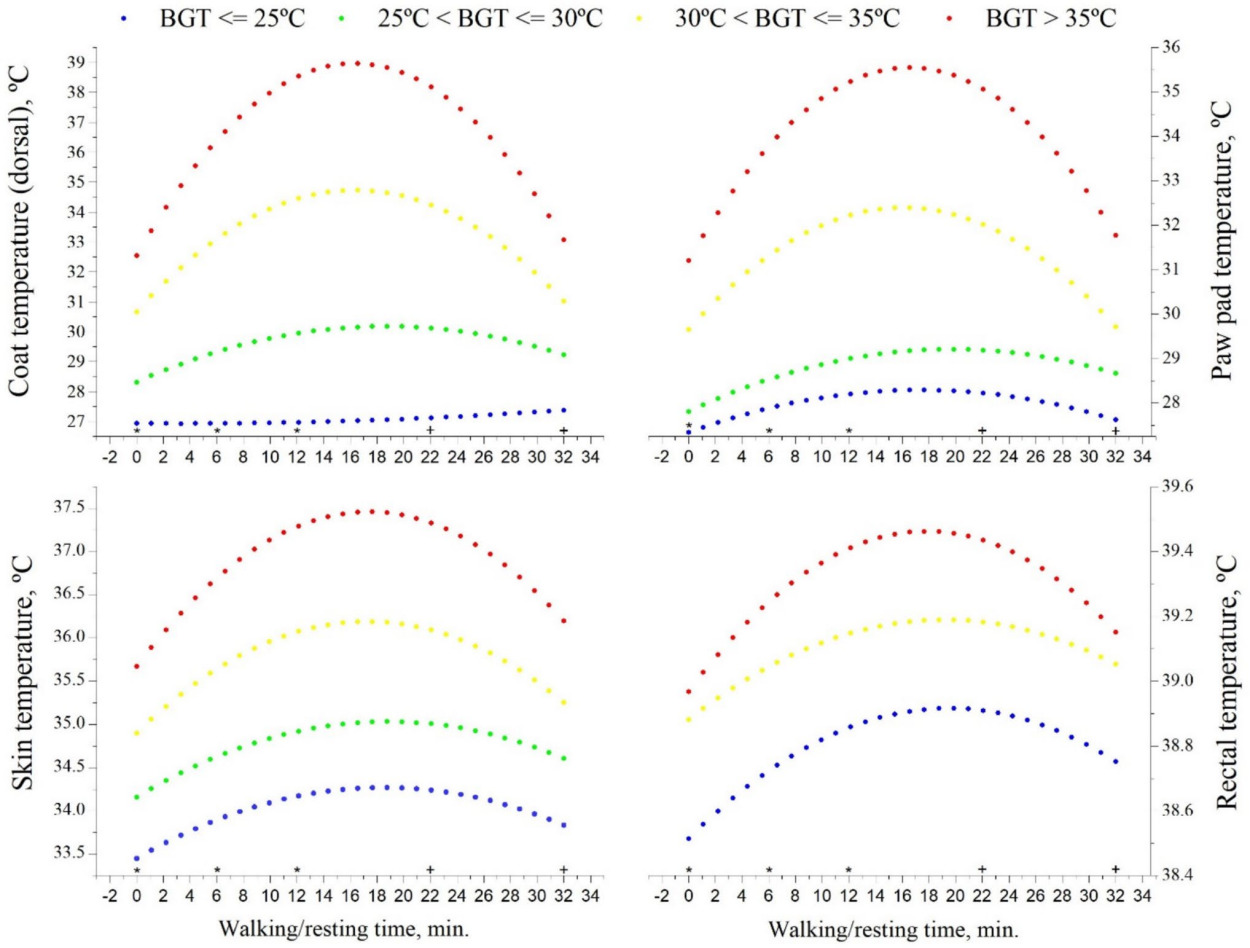
Body temperatures of dogs (n = 5) predicted every minute, as a function of walking/resting time (X) and heat load experienced (Y). *Values observed/measured during walking phase. + Values observed during the recuperation phase

Overall, thermoregulatory responses increased with heat load and walking duration, and decreased progressively during the recovery phase (Fig. 2). The hair-coat temperature remained stable when the BGT class was below 25 °C; however, it increased linearly as radiant heat load increased during 12 min walk, reaching a peak of ~ 39 °C under conditions of BGT class above 35 °C. During the 20 min recovery phase, irrespective to the heat load experienced by dogs, the hair-coat temperature returned to its baseline value (i.e., walking time 0)(Fig. 2). The skin temperature increased with both walking duration and radiant heat load, but it did not return to its initial value during the recovery phase (Fig. 2).

The paw pad temperature increased throughout the walk only when the BGT was higher than 30 ºC (i.e., BGT classes 3 and 4); for all other BGT classes, the paw pad temperature returned to its initial value during the recovery phase (Fig. 2). The rectal temperature varied between 38 ºC and 39 ºC. During walks within the BGT class 1, the rate of increase in the rectal temperature was close to 0.01 ºC min^−1^ whereas in BGT class 4, this rate increased to 0.03 ºC min^−1^. For all other BGT classes, the rectal temperature also did not return to its initial value during the recuperation period (Fig. 2).

**Fig. 3:**
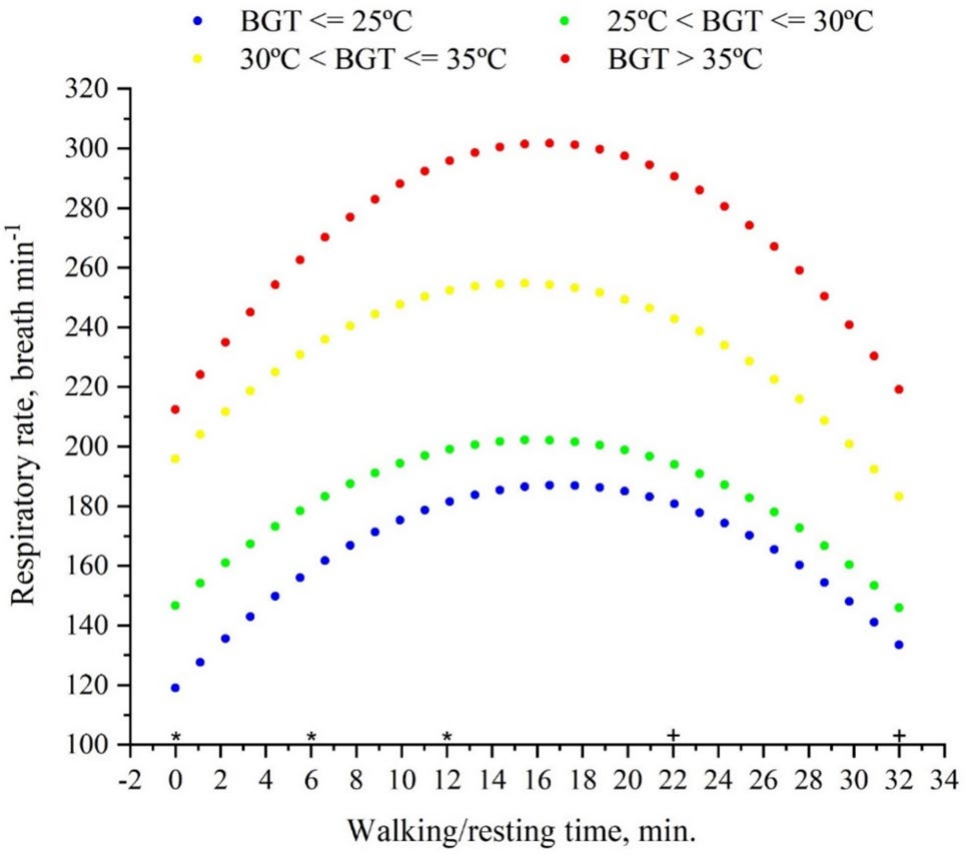
Respiratory rate of dogs (n = 5) predicted every minute, as a function of walking/resting time (X) and heat load experienced (Y). *Values observed/measured during walking phase. + Values observed during the recuperation phase

The respiratory rate increased with walking duration and radiant heat load (Fig. 3). In the walks conducted under BGT class 4, the respitory rate peaked at approximately 300 breaths min⁻^1^ (5 breaths s⁻^1^), as predicted by the model, and returned to its initial value during the recovery phase (Fig. 3).

**Fig. 4:**
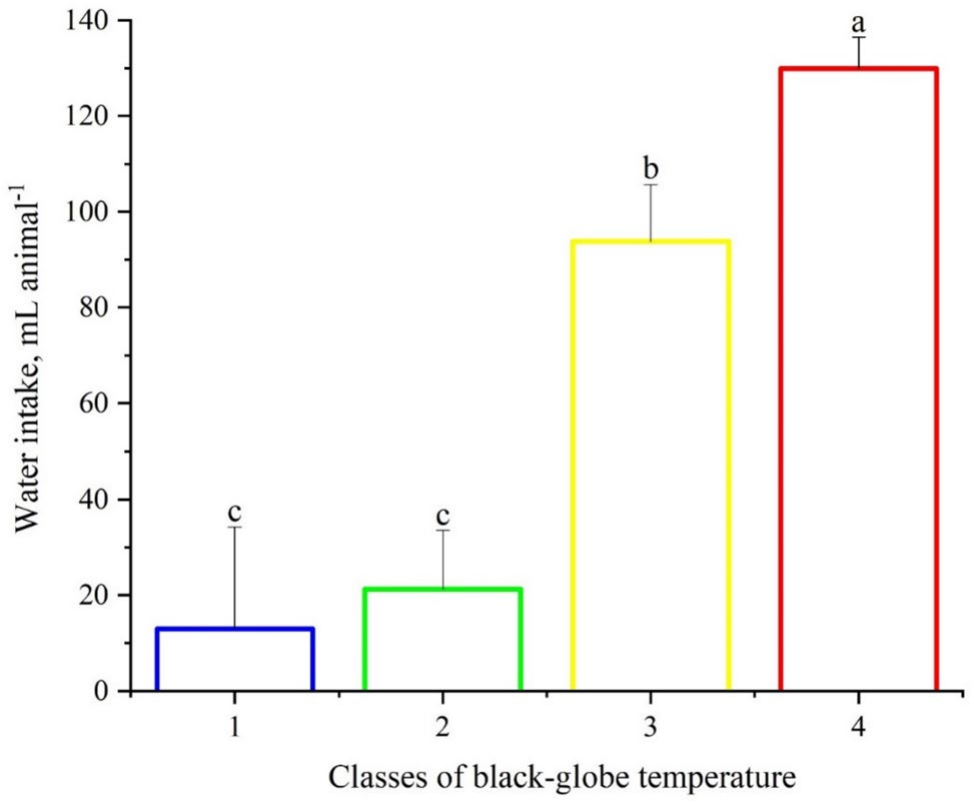
Water intake (mL) of dogs (n = 5) during the rest period post-walking in relation to BGT classes. Means with different letters show significant statistical difference between them, according to the Tukey test at 5% probability. First class (1): 25 °C ≥ BGT, second class (2): 30 °C ≤ BGT > 25 °C, third class (3): 35 °C ≤ BGT > 30 °C, fourth class (4): BGT > 35 °C

There was no difference in water consumption during the rest period after dog walks conducted under BGT classes 1 and 2. However, water intake increased after walks conducted under BGT class 3 compared to classes 1 and 2, and was highest in class 4 compared to all other BGT classes (Fig. 4). The minimum water consumed was 10 mL when BGT was ≤ 25 °C, and the maximum was 215 mL when BGT was > 35 °C, representing an increase of over 200 mL from BGT class 1 to class 4.

### Critical heat stress thresholds for dogs based on the TSI

**Fig. 5:**
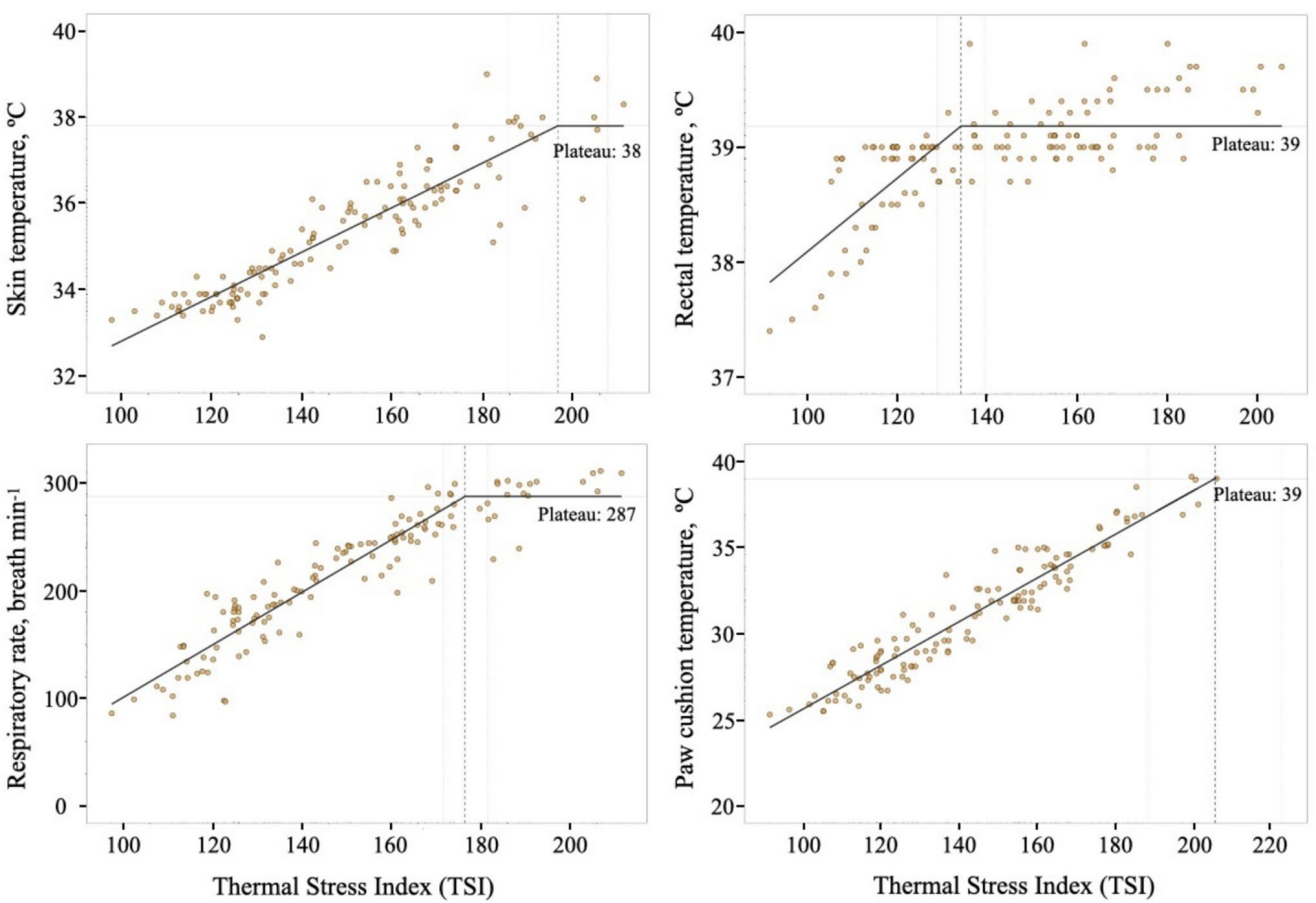
Values of skin temperature, rectal temperature, respiratory rate, and paw cushion temperature as a function of the Thermal Stress Index (TSI)

The TSI ranged from 90 to 220. The plateau for skin temperature, rectal temperature, respiratory rate and paw pad temperature were TSI ≈ 195 (38 °C), TSI ≈ 135 (39 °C), TSI ≈ 180 (287 breaths min⁻^1^) and TSI ≈ 205 (39 °C), respectively (Fig. 5).

## Discussion

This study is the first to determine critical heat stress thresholds for domestic dogs during outdoor walks in tropical conditions. During walks, dogs experienced black-globe temperatures in the sun of up to 50 °C. The results revealed that regardless of the high heat load experienced during the walks, dogs kept their rectal temperature within a narrow range (38—39 ºC) and below the critical limits for hyperthermia (i.e., > 41ºC). However, as black-globe temperature in the sun increased from 25 °C to values above 35 °C, even during short walking sessions, dogs exhibited marked increases in skin temperature, indicating enhanced peripheral vasodilation (+ 21.6%), and panting activity, which increased by approximately sevenfold compared to lower heat load conditions, thereby indicating enhanced reliance on respiratory evaporative heat loss. The marked increase in panting was accompanied by an approximately 20-fold increase in post-walk water intake, indicating a pronounced physiological demand for rehydration. The autonomic responses and body temperatures of dogs were summarized into a single variable, the TSI, which was better predicted by the combined effects of black-globe temperature in the shade and in the sun, in addition to ambient air temperature. The TSI ranged from 90 to 220. The respiratory rate increased linearly from 100 breaths min^−1^ (1.66 breaths s^−1^) to 287 breaths min^−1^ (4.80 breaths s^−1^), as TSI rose from 100 to ~ 180, beyond which it plateaued despite further increases in TSI. Although we did not measure additional respiratory mechanics (e.g., tidal volume and minute ventilation), evidence from the classical work of Crawford (1962) suggests that panting frequency tends to approach the natural resonant frequency of the respiratory system, at which pulmonary ventilation is maximized with minimal mechanical effort. Increases in respiratory rate beyond this optimal range possibly yield limited gains in ventilatory efficiency. Therefore, we believe that the respiratory rate plateau at higher TSI values observed in the present study reflects a progressive constraint in the capacity to further increase mass and heat transfer through the respiratory tract.

Rectal temperature is one of the main heat strain indicators of homeothermic animals (Lewis and Foster 1976), and dogs are considered moderately hyperthermic when it approaches 40 °C (Zanghi 2016). In the present study, the rectal temperature remained within a relatively narrow range during most walking sessions, although occasional increases above 40 °C were observed under higher heat loads. However, the apparent stability of rectal temperature should not be interpreted as an absence of thermal stress. The rate of increase in rectal temperature was markedly different according to the experienced heat load, rising from approximately 0.01 °C min⁻^1^ under lower radiant heat load conditions to about 0.03 °C min⁻^1^ when black-globe temperature exceeded 35 °C. If such rates were sustained during prolonged exposure, the negative physiological implications could be substantial for the dogs. For example, starting from a baseline of approximately 38 °C, a rate of increase of 0.03 °C min⁻^1^ would result in an increase of about 2 °C within roughly one hour, potentially pushing animals into moderate hyperthermia. Therefore, the maintenance of rectal temperature within a narrow range during the relatively short walking sessions evaluated here likely reflects active thermoregulatory compensation, rather than low thermal challenge. These findings suggest that under prolonged exposure to similar environmental conditions, compensatory mechanisms could become insufficient to prevent progressive heat storage.

The thermoregulatory responses observed during walking were also consistent with progressive limitations of sensible heat exchange pathways. Hair-coat surface temperature increased markedly under elevated radiant heat load, reflecting substantial absorption of short-wave solar radiation. In the present study, hair-coat surface temperatures frequently exceeded both ambient air temperature and black-globe temperature measured in the shade, particularly under higher radiant heat load. This indicates that a positive thermal gradient between the animal's surface and the surrounding environment persisted, allowing net outward sensible heat transfer through convection and long-wave radiation. However, as solar radiation intensifies, the animal absorbs large amounts of short-wave radiation at the coat surface, thereby increasing the heat load that must be transferred to the environment. Under such conditions, sensible heat transfer may become insufficient to fully offset the combined effects of metabolic heat production and radiative heat gain.

Skin temperature increased and reached a plateau at approximately 38 °C (Fig. 5), indicating activation of peripheral vasodilation. This response enhances heat transfer to the environment and represents an important thermoregulatory adjustment (Almeida et al. 2020). However, the capacity for heat dissipation through peripheral vasodilation in dogs is inherently limited when compared with species that rely heavily on cutaneous evaporation. Because dogs exhibit minimal sweating across most of the body surface and possess relatively limited funcional eccrine sweat grand activity, their ability to modulate skin temperature through cutaneous evaporation is limited (Hammel et al. 1958; Bruchim et al. 2017). The relatively modest range of variation in the skin temperature observed in this study, even under high heat load, supported this statement.

When sensible heat transfer mechanisms become insufficient to dissipate metabolic and absorbed heat, dogs activate panting as a primary autonomic thermoregulatory response. This autonomic response results in rapid shallow breathing, characterized by increments in respiratory rate fallowed by decreases in tidal volume, a process that enhances evaporative heat loss from the upper respiratory tract. In the present study, respiratory rate of dogs during walking reached respective mean and maximum values of 206 ± 65.3 breaths min⁻^1^ and 287 breaths min⁻^1^, which were substantially higher than values reported for resting shaded dogs (Antink et al. 2019), indicating strong activation of this thermoregulatory pathway under elevated radiant heat load. Under controlled climatic conditions, panting frequency in heat-exposed dogs can exceed 350 breaths min⁻^1^ (Higgins and Iampietro 1967), and such elevations were associated with progressive reductions in blood CO₂ concentration and increases in blood pH, indicating thermally induced respiratory alkalosis. Increases in respiratory rate do not indefinitely translate into proportional gains in respiratory evaporative heat loss. Mechanical analyses of panting demonstrate that pulmonary ventilation increases with respiratory rate only up to approximately 5 cycles s⁻^1^, beyond which further increases provide little additional cooling effects due to reductions in tidal volume and mechanical constraints of the respiratory system (Crawford 1962).

Within this physiological framework, the proposed critical thermal stress threshold identified in this study (TSI ≈ 170) (Fig. 5) represents the point at which dogs appear to reach a high level of thermoregulatory activation, characterized by intense panting, enhanced peripheral vasodilation, and high rates of heat storage.. Beyond this threshold, sustained homeothermy is likely to become challenging under conditions of prolonged walks. Experimental evidence suggests that once respiratory frequency approaches its upper physiological range, additional increases in ventilation are achieved primarily through increases in tidal volume rather than respiratory rate (Kanter 1959; Higgins and Iampietro 1967), a shift associated with increased physiological cost, respiratory water loss, and progressive reduction in thermoregulatory safety margin. The marked increase in post-walk water intake observed under higher heat loads in the present study supports this interpretation, indicating increaments on body water demand associated with sustained panting and respiratory evaporative water loss. If environmental heat load and metabolic heat production continue to exceed the animal’s capacity for effective heat dissipation, compensatory mechanisms may become insufficient to sustain homeothermy over prolonged periods, particularly if dehydration begins to limit evaporative cooling efficiency and circulatory performance. Under such conditions, susceptibility to heat-related illness, including heat stroke, increases substantially (Teichmann et al. 2014; Baltzer et al. 2012). Thus, a TSI value near 170 can be interpreted as the approximate upper limit of effective thermoregulatory compensation during outdoor walking under hot and sunny conditions. Importantly, under the conditions evaluated in this study, dogs were able to maintain physiological stability, indicating that these thresholds reflect increased thermoregulatory effort rather than thermoregulatory failure.

These findings have direct implications for dog welfare, particularly in tropical and subtropical regions where outdoor activities frequently occur under conditions of high radiant heat load Some limitations of the present study should be acknowledged. Wind speed was not explicitly incorporated into the index and may influence convective and evaporative heat exchange under field conditions. The sample size was relatively small and consisted primarily of mixed-breed dogs adapted to tropical environments. Given the high diversity of the domestic dog population, broader representation of breeds and morphotypes is necessary for comprehensive assessment of heat stress responses across different anatomophysiological profiles and heat load. Brachycephalic dogs, for example, are known to exhibit higher respiratory rates and greater susceptibility to heat stress compared with non-brachycephalic dogs (Davis et al. 2017). Additionally, coat characteristics, body condition, and physical fitness may influence thermoregulatory capacity in different environmental contexts. Futher studies should therefore incorporate a wider range of breeds and environmental scenarios to refine the applicability of the proposed index.

## Conclusion

Walking with domestic dogs under TSI values exceeding approximately 170—190 may impose substantial thermoregulatory strain and should be approached with caution, as it reflects a high level of thermoregulatory effort, particularly during prolonged exposure.

## Data Availability

Not applicable.

## Declarations

Nothing to declare.
